# Occurrence of health-compromising protozoan and helminth infections in tortoises kept as pet animals in Germany

**DOI:** 10.1186/s13071-018-2936-z

**Published:** 2018-06-18

**Authors:** Malek J. Hallinger, Anja Taubert, Carlos Hermosilla, Frank Mutschmann

**Affiliations:** 1 Institute of Parasitology, Justus Liebig University Giessen, Giessen, Germany; 2 Exomed GbR, Berlin, Germany

**Keywords:** Exotic pets, Tortoise, Herpetology, Pet reptiles, Reptile medicine, Tortoise endoparasites

## Abstract

**Background:**

Exotic reptiles such as tortoises, have become increasingly common domestic pets worldwide and are known to host different gastrointestinal parasites. Some of these parasites bear zoonotic potential. In the present survey, we parasitologically examined tortoise faecal samples (*n* = 1005) from 19 different species held as pets in private German households and German zoological gardens.

**Methods:**

Saline faecal smears were used to generate prevalence data for potentially health-compromising gastrointestinal parasites. In addition, we performed complete parasitological dissections of dead tortoises (*n* = 49) to estimate endoparasite burdens precisely.

**Results:**

Analysed tortoise faecal samples contained a broad spectrum of endoparasites. We detected ten taxa of endoparasites; oxyurid nematodes (e.g. *Tachygonetria* spp.) were the most prevalent parasites in faecal samples (43.18%), followed by ascarids (*Angusticaecum* spp.) (0.01%), *Hexamita* spp. (0.007%), *Balantidium* spp. (0.007%), trichomonads (0.004%), *Strongyloides* spp. (0.003%), *Entamoeba* spp. (0.005%), *Hartmanella* spp. (0.001%), *Blastocystis* spp. (0.002%)*,* heterakids (0.001%) and *Trimitus* spp. (0.001%)*.* Additionally, we investigated dead tortoise individuals (*n* = 49; of 10 different species) for aetiological diagnosis and estimation of endoparasite burden. Of these individuals, 38 (77.6%) were infected with parasites and 14 (28.6%) of them died most probably due to severe parasitic infection. Oxyurid infections correlated positively with calcium deficiency and metabolic bone disease (MBD) as well as nephrosis/nephritis, mainly occurring in juvenile tortoises (< 5 years of age).

**Conclusions:**

The saline faecal smear technique proved to be efficient in detecting different metazoan and protozoan parasite stages in tortoise faeces. The prevalence of oxyurid infections was particularly high. In combination with pathological findings in clinical oxyuridosis obtained from necropsied animals, our findings call for further, detailed investigations on pathogenesis and immunology of oxyurids in pet reptiles. Coprological analyses for parasite detection should be mandatory before tortoises are transferred to a new owner, animal group, or public and private enclosures such as zoos. We advocate for regular health screenings in pet tortoises and, if parasitic infections are diagnosed, adequate medication or alternative hygiene management should be considered to improve and maintain individual and population health.

**Electronic supplementary material:**

The online version of this article (10.1186/s13071-018-2936-z) contains supplementary material, which is available to authorized users.

## Background

Reptiles have become increasingly popular domestic pets worldwide, and significant reptile welfare problems are linked to international pet trade [1, 2]. While several reptile species obtainable as pet animals are bred successfully in captivity, other species are already taken from wild populations, reducing wild population sizes, and are used for pet trading or breeding [1–3]. Therefore, many tortoise species are critically endangered in the wild worldwide [1, 4, 5]. At the same time, tortoises are becoming increasingly popular domestic pets [4, 5]. Exotic reptiles originating from the wild (including tortoises), in particular, are often infected with a variety of different invasive parasites, harbouring a broad spectrum of endogenous parasites including diverse species of protozoans, nematodes, cestodes, pentastomids, acanthocephalans and trematodes [6–10]. Tortoises specifically are not exclusively infected by gastrointestinal parasites such as oxyurids, ascarids and protozoans, but also carry diverse ectoparasites. Despite tortoises’ popularity as pets, very little is known about the ectoparasite fauna affecting them in captivity [11] and thus needs further attention.

Reptiles held in captivity show higher prevalences and more efficient transmission for different monoxenous parasitoses than wild reptiles, including those caused by metazoan as well as protozoan parasites [12, 13]. Plausible explanations for this phenomenon are mainly linked to direct life-cycles together with high tenacity of certain reptile exogenous parasitic stages, i.e. eggs, larvae, cysts and oocysts [12, 14]. Pathogenicity of reptile parasitoses varies due to husbandry conditions, and poor husbandry hygiene can lead to clinically relevant massive parasitic burdens in a terrarium due to frequent reinfections [12]. Additionally, captivity stress can exacerbate existing parasitic infection as well as negatively impact on the host innate immune system [12, 15]. Endoparasites can cause different clinical symptoms depending on parasite species and life-cycle, the age, sex, and health status of the animal host, housing conditions, and degree of infection [12, 16]. In severe parasitoses, developmental disorders or even mortality can occur. Additionally, limited space and reinfection or superinfection can be critical for the survival of a captive-held reptile [3, 12]. Even more importantly, several reptile-borne parasites and bacteria exhibit zoonotic potential. For example, *Salmonella enterica* and *S. bongori*, their subspecies and serovariants [12, 17, 18], pentastomids, such as *Porocephalus* spp. and *Armillifer* spp. in American and African snakes and *Raillietiella* spp. in snakes and geckos [8, 18], cestodes [19, 20], nematodes, e.g. *Trichinella* spp. in crocodiles [21] and *Cryptosporidium* spp. [22, 23] have been discussed as relevant anthropozoonotic parasites. Reptile-borne anthropozoonoses are also common in countries where reptiles are exploited for consumption as well as traditional medicinal and ethnic practices [8, 21]. There, common infection sources are the ingestion of raw or insufficiently cooked reptile meat, the usage of reptile meat as compresses to heal wounds or abscesses and drinking of exogenous water with contaminated parasite life-cycle stages [8, 19, 20].

In the case of captive tortoises, parasitological investigations performed recently in other European countries [13, 14, 16, 24, 25] revealed high and strongly varying prevalence (43–82%) of gastrointestinal parasites in tortoises. The assessment methods in parasitological studies on tortoises varies strongly, making inter-study comparisons challenging. Some studies focused on dead or sick animals [13]. A study by Pasmans et al. on captive tortoises in Germany showed parasite infection prevalence but did not investigate commensalism and parasite-derived pathogenicity nor tortoise species, sex, age or nutrition status [12, 22]. Other reports focused on therapy options and prevalence [26], whereas others focused on oxyurid-infected tortoises [27, 28]. Further tortoise studies exclusively examined imported, sick or dead animals [12] without addressing current parasitic prevalence in pet tortoise populations.

Therefore, the aim of the current study is to provide new data on the prevalence of gastrointestinal parasites within the captive pet tortoise population in Germany and to discuss potential impact of these parasites on animal and public health.

## Methods

### Faecal samples

To obtain representative data for the prevalence of the gastrointestinal endoparasites affecting pet tortoises in Germany, we performed a stratified randomized sample of the entire captive population of Germany. We performed coprological analyses of a total of 1005 faecal samples, collected from 19 different tortoise species (Table 1), from November 2015 to December 2016. Analyzed faecal samples originated from animals either owned privately or held in German zoos, and were posted to the Exomed Laboratory in Berlin, Germany. We examined all samples by using saline faecal smears for general parasitological diagnosis.

**Table 1:** Examined faecal samples of tortoises and origin of sender (total *n* = 1005)

Tortoise species	Common name	No. examined	Origin (private/vet/zoo)
*Testudo hermanni*	Hermann’s tortoise	597	491/105/1
Unkown species	–	169	143/26/0
*T. graeca*	Common tortoise	65	42/21/2
*T. horsfieldii*	Horsefield’s tortoise	61	38/23/0
*T. marginata*	Marginated tortoise	50	43/7/0
*Stigmochelys pardalis*	Leopard tortoise	11	6/4/1
*Centrochelys sulcata*	African spurred tortoise	10	8/1/1
*Geochelone elegans*	Indian star tortoise	10	8/2/0
*Chelonoidis carbonaria*	Red-footed tortoise	7	4/1/2
*Aldabrachelys gigantea*	Aldabra giant tortoise	5	3/1/1
*T. kleinmanni*	Egyptian tortoise	4	3/0/1
*Malachoserus tornieri*	Pancake tortoise	4	1/1/2
*Geochelone platynota*	Burmese star tortoise	3	0/0/3
*Astrochelys radiata*	Radiated tortoise	3	1/1/1
*Indotestudo elongata*	Elongated tortoise	2	2/0/0
*Pyxis arachnoides*	Spider tortoise	2	2/0/0
*Kinixys bellinia*	Bell’s Hinge-backed tortoise	1	1/0/0
*Manouria impressa*	Impressed tortoise	1	1/0/0
*Chersina angulata*	Angulate toroise	1	1/0/0

Due to controversial issues recording tortoise phylogenetic position of Chelonia [11, 29], we here investigated exclusively faecal samples from terrestrial and major-herbivore species [30–35]. All analysed tortoises belonged to the family Testudinae [11, 29] and contained mainly European species (773 out of 1005 tortoises). Additionally, Exomed GbR clients were asked to provide a printed form delineating animals’ signalment (i.e. species, sex, age), husbandry conditions (e.g. origin of tortoise, time in owner’s possession, individual/group housing, indoor/outdoor enclosure), previous parasitological examinations, and any anthelminthic treatments, labelled with the same reference number as corresponding faecal sample.

To ensure that all samples could be analyzed within 24 h of arrival, we encouraged tortoise owners to sample only fresh faeces, and to send these samples to our laboratory immediately. On arrival, samples were randomized, numbered, and stored at 4 °C. We conducted a macroscopic examination of each faecal sample with a direct wet (saline) preparation according to Cooper [36]. By mixing 10 g of faeces at a ratio of 1:1 with 0.9% saline solution, a uniform solution was created. This solution allowed us to simultaneously identify different stages of both helminths and protozoans. Fifty millilitres of this solution was carefully placed on glass coverslides and gently covered by glass cover slips (22 × 22 mm). Examination was performed at 100× and 400× magnification under a light microscope (Axio Imager M1, Zeiss, Jena, Germany). Counts of metazoan parasitic eggs and of protozoan trophozoites, cysts and oocysts were estimated.

We identified nematode eggs (e.g. oxyurids, ascarids and strongylids) and protozoan trophozoites, cysts and oocysts (e.g. *Balantidium*, *Nyctotherus*, *Hexamita* and *Cryptosporidium*) based on morphological/morphometric descriptions reported elsewhere [3, 6, 7, 28, 37]. A faecal sample was categorized as ‘positive’, when at least one stage of a potentially health-compromising described endoparasite was found. A sample containing exclusively non-pathogenic or facultative parasitic genera (i.e. *Balantidium*, *Nyctotherus*, *Blastocystis*, *Trimitus* and other flagellated protozoans) was classified as ‘negative’, unless it contained an extraordinary high number of flagellates, trophozoites or cysts according to previous reports [12, 16, 37, 38].

### Tortoise necropsies

We necropsied a total of 49 tortoise corpses of at least eight different tortoise species at the Exomed Laboratory (see Table 2). We collected anamnestic data as described in the previous section for faecal samples. External examinations and necropsies were performed according Rataj et al. [13]. Additionally, we performed pathohistological examinations. The carcasses of most of tortoises had been freshly frozen prior to shipping to the Exomed Laboratory for *post-mortem* examination. The digestive tract was systematically examined for the presence of endoparasites. Intestinal contents were examined further by using saline faecal smears. Morphological identification of endoparasites was conducted under a light microscope (Axio Imager M1, Zeiss) equipped with a digital camera (AxioVision Software).

**Table 2:** Performed necropsies of tortoises, tortoise species and origin of sender (*n* = 49)

Tortoise species	Common name	No. examined	Origin (private/vet/zoo)
*T. hermanni*	Hermann’s tortoise	20	17/2/1
Unkown species	–	7	4/3/0
*T. horsfieldii*	Horsefield’s tortoise	5	5/0/0
*T. graeca*	Common tortoise	5	2/3/0
*Stigmochelys pardalis*	Leopard tortoise	5	5/0/0
*T. kleinmanni*	Egyptian tortoise	2	2/0/0
*Pxyis arachnoides*	Spider tortoise	2	0/0/2
*Centrochelys sulcata*	African spurred tortoise	1	1/0/0
*Geochelone chilensis*	Argentine tortoise	1	1/0/0
*Manouria impressa*	Impressed tortoise	1	1/0/0

### Microbiology

If requested by the owner, we performed selectively bacterial and fungal isolation. Swabs of faeces or of the coelom were inoculated on sheep blood agar (5%), MacConkey agar and Sabourand dextrose agar (SDA) (BioMerieux, Charbonnier les Bains, France), respectively. Bacterial isolates were identified by Gram straining, oxidase and katalase test, as well as a commercial API 20E/NE kit (BioMerieux) as described for ectothermic vertebrates, such as fish [39].

## Results

### Geographical origin of faecal samples, tortoise species and occurring endoparasites

**Fig. 1:**
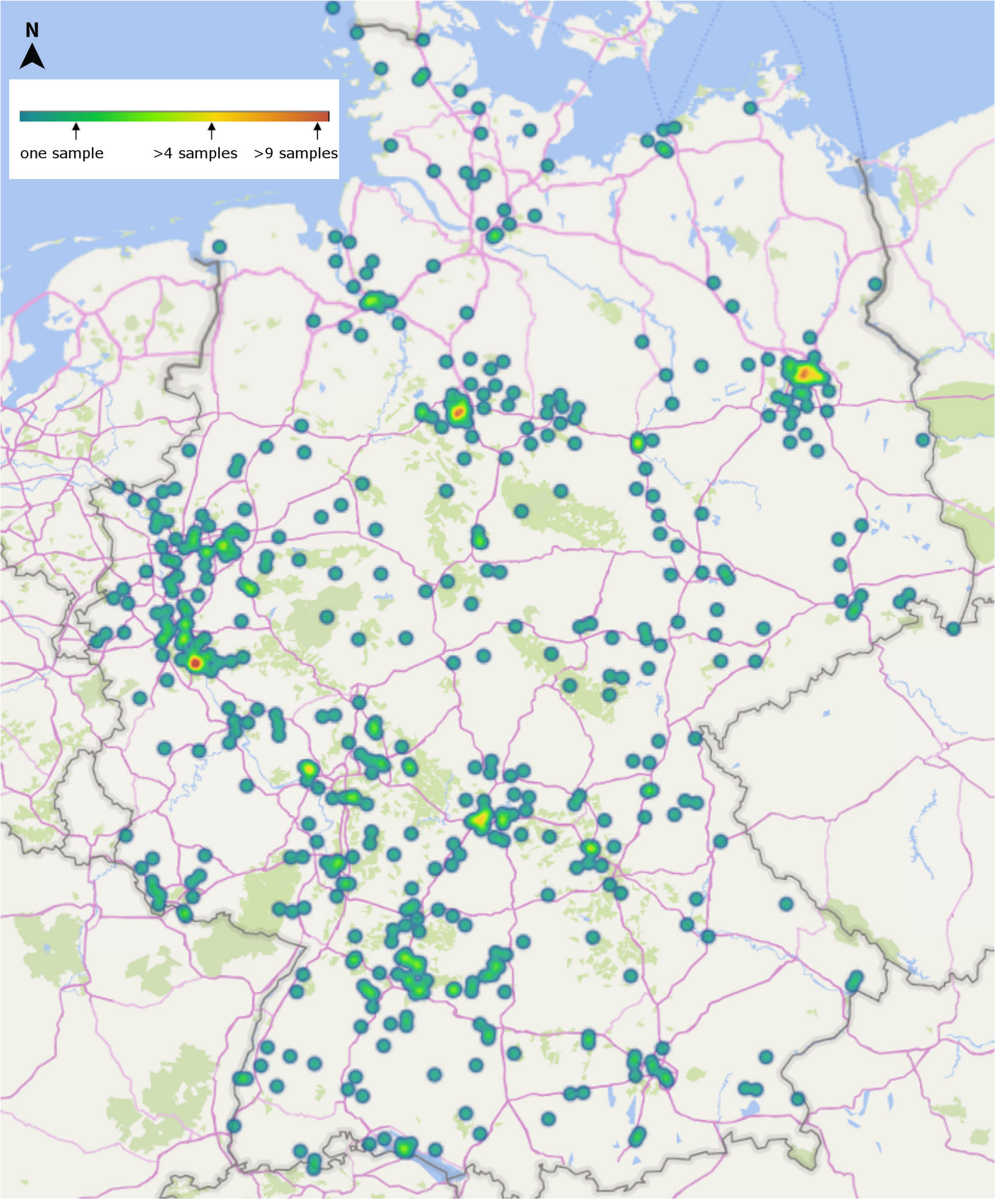
Geographical origin and number of faecal samples according to postcode districts in Germany

**Fig. 2:**
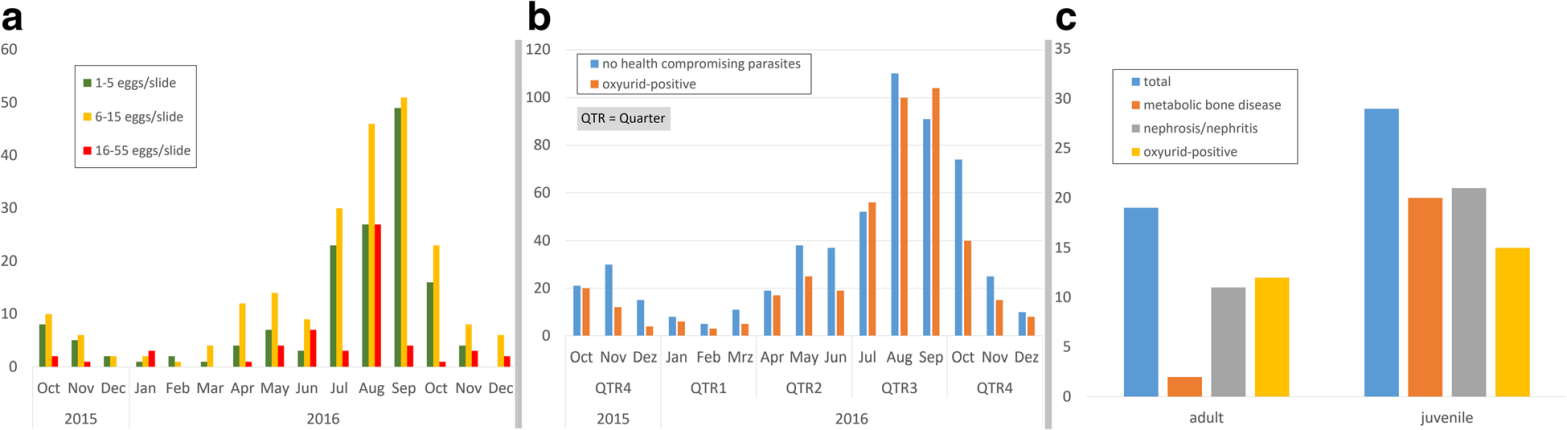
Seasonal distribution of parasite-positive and -negative faecal samples and age distribution of examined tortoises during the study period. **a** Each bar represents the number of nematode eggs found in parasite-positive faecal smears of tortoises during the years 2015 and 2016. **b** Each bar represents the number of negative and oxyurid-positive tortoise samples analysed during the years 2015 and 2016. **c** Each bar illustrates number of clinical findings in analysed tortoises according to their ages [juvenile (< 5 years) and adults]. MBD appeared more frequently in juvenile tortoises, while nephrosis or nephritis also affected the cholecalciferol production (vitamin D3) in the kidney

Geographical origin and tortoise species included in this parasitological survey are listed in Fig. 1 and Table 1. Within this survey, 19 different tortoise species were examined. The origin of the samples is visualized on a geographical map of Germany (Fig. 1). In total, five different metazoan parasite species, all belonging to the class Nematoda, were recorded (Tables 3 and 4). Among these nematodes, oxyurid eggs were the most common helminth ova identified in 43.2% of the samples from 14 out of 19 different tortoise species. In contrast, only 12 samples (0.1%) contained *Angusticaecum* spp. eggs, three samples contained *Strongyloides* spp. eggs (0.003%) and one sample contained heterakid eggs (0.001%). A complete list of parasite species identified in the study is shown in Table 3. All nematode species reported here have pathogenic relevance for tortoises, and a negative impact on individual/population health. Seasonal oxyurid infection rates differed significantly (Chi-square test: *χ*^2^ = 10.97, *df* = 1, *P* < 0.001), being higher in winter (35.53%) than in summer (46.66%) (see Fig. 2a, b and Table 5). Also, oxyurid infection rates varied with age (Chi-square test: *χ*^2^ = 11.73, *df* = 1, *P* < 0.001), such that individuals older than five years were infected less frequently (38.57%) than juveniles younger than five years of age (52.04%). No significant correlation in oxyurid burdens could be detected in relation to other factors such as tortoise species, sex, group size and maintenance conditions (Table 5).

**Table 3:** Number and percentage of positive tortoises regarding infection with gastrointestinal endoparasites (total *n* = 1005; 459 positive and 546 negative)

Parasite species	No. of positive (%)	Host species (*n*)
Oxyurid nematode (*Tachygonetria* sp.)	434 (43.2)	*T. hermanni* (248); unknown species (94); *T. marginata* (28); *T. graeca* (21); *T. horsfieldii* (19); *Stigmochelys pardialis* (5); *Centrochelys sulcata* (5); *Geochelone elegans* (4); *Chelonoidis carbonaria* (2); *T. kleinmanni* (2); *Pyxis arachnoides* (2); *Malacochersus tornieri* (1); *Chersina angulate* (1); *Aldabrachelys gigantea* (1); *Astrochelys radiata* (1)
Ascarid nematode (*Angusticaecum* sp.)	12 (0.01)	*T. hermanni* (5); unknown species (5); *Stigmochelys pardalis* (1); *C. sulcata* (1)
*Hexamita* sp.	7 (0.007)	*T. hermanni* (5); *Geochelone elegans* (1); *Chelonoidis carbonaria* (1)
*Balantidium* sp (cysts)	7 (0.007)	*T. hermanni* (*5*); *C. sulcata* (1); *Geochelone platynota* (1)
*Trichomonas* sp.	4 (0.004)	*T. hermanni* (4)
Strongyloid nematode (*Strongyloides* sp.)	3 (0.003)	unknown species (*2*); *Geochelone elegans* (1)
*Entamoeba* sp. (cysts)	5 (0.005)	*T. hermanni* (1); *Geochelone elegans* (1); *Chelonoidis carbonaria* (1); unknown species (1)
Heterakid nematodes	1 (0.001)	*Geochelone elegans* (1)
*Hartmanella* sp. (*Limax* amoeba)	1 (0.001)	*Geochelone elegans* (1)
*Blastocystis* sp.	2 (0.002)	*T. hermanni* (1); *Geochelone elegans* (1)
Facultative pathogen flagellates (*Trimitus* sp.)	2 (0.002)	*T. horsfieldii* (1); *Geochelone elegans* (1)

**Table 4:** Number and percentage of positive tortoise corpses regarding to infestation with potentially health-compromising endoparasites (total *n* = 49 / 38 positive and 7 different gastrointestinal parasites detected)

Parasite species	No. of positive (%)	Host species (*n*)
Oxyurid nematode (*Tachygonetria* sp.)	28 (57.14)	*T. hermanni* (14); unknown species
*Balantidium* sp. (cysts)	3 (6.12)	*T.horsfieldii* (3); *T. graeca* (3); *T. kleinmanni* (1); *Stigmochelys pardalis* (1); *G. sulcata* (1)
*Hexamita* sp.	2 (4.08)	*Manouria impressa* (1); *Pyxis arachnoides* (1)
*Blastocystis* sp.	2 (4.08)	*Pyxis arachnoides* (1); unknown species (1)
*Entamoeba* sp. (cysts)	2 (4.08)	*Pyxis arachnoides* (1); unknown species (1)
Heterakid nematodes	2 (4.08)	*Stigmochelys pardalis* (2)
Strongyloid nematode (*Serpinema* sp.)	1 (2.04)	unknown species (1)
*Proataracis* sp.	1 (2.04)	*T. hermanni* (1)

**Table 5:** Statistical analysis of specific risk factors for oxyuridosis from data obtained by pet owners

Factor	Sample size	No. of negative (%)	No. of oxyurid-positive (%)	Chi-square^a^	*P*-value (two-sided)
Sender	991			16.28	< 0.001^b^
Private	798	426 (53.4)	372 (46.6)		
Veterinary doctor	193	134 (69.4)	59 (30.6)		
Tortoise species	452			2.97	0.086
*T. hermanni*	402	222 (55.2)	180 (44.8)		
*T. graeca*	50	34 (68.0)	16 (32.0)		
Tortoise sex	725			1.43	0.231
Male	253	150 (59.3)	103 (40.7)		
Female	472	258 (66.7)	214 (45.3)		
Tortoise age	644			11.73	< 0.001^b^
Juvenile; < 5a	294	141 (48.0)	153 (52.0)		
Adult; > 5a	350	215 (61.4)	135 (38.6)		
Group size	765			0.99	0.321
Group facility	648	350 (54.0)	298 (46.0)		
Single enclosure	117	69 (60.5)	48 (41.0)		
Keeping conditions	111			0.32	0.571
Free- range	94	62 (66.0) /	32 (34.0)		
Terrarium	11	10 (58.8)	7 (41.2)		
Sampling season				10.968	< 0.001^b^
October-March	318	205 (64.5)	113 (35.5)		
April-September	688	367 (53.3)	321 (46.7)		

^a^Chi-square test, *df* = 1, α = 0.001; critical *χ*^2^ value = 10.82)

^b^Significant difference

**Fig. 3:**
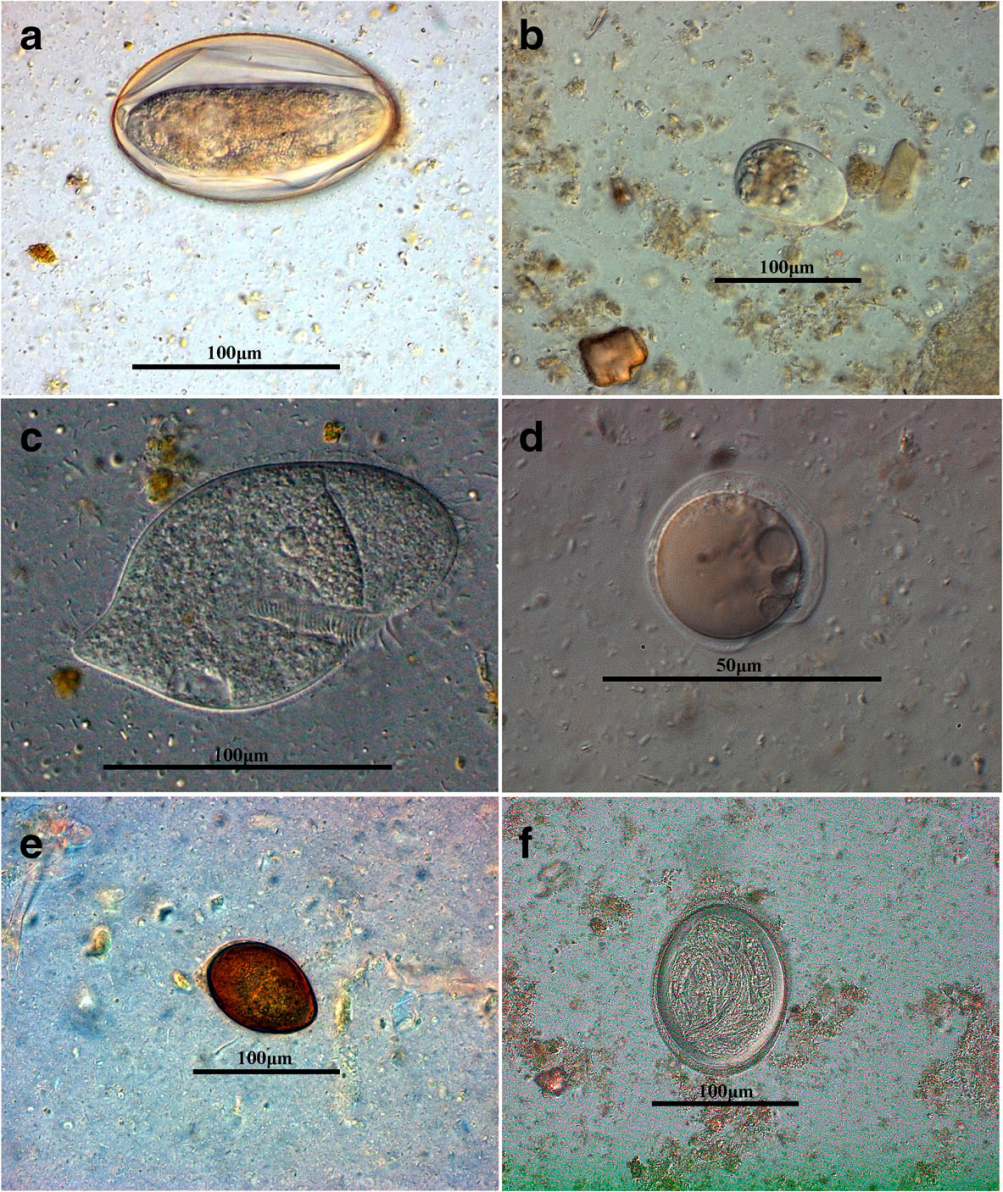
Light microscopy photomicrographs of parasite life-cyste stages found in faecal samples of German pet tortoises. **a** Oxyurid egg from a Marginated tortoise (*Testudo marginata*). **b** Iodide-stained *Entamoeba* trophozoite from a Hermann tortoise (*Testudo hermanni*). **c** Ellipsoidal-shaped *Balantidium* sp. trophozoite with a long cytostome and oral cavity, from a Hermann tortoise (*T. hermanni*). **d** *Blastocystis* sp. cyst from a Hermann tortoise (*T. hermanni*). **e** *Nyctotherus* sp. cyst in the faeces of an Indian Star tortoise (*Geochelone elegans*). **f** *Angusticaecum* sp. egg from a Hermann tortoise containing infectious L1 (*T. hermanni*)

The diversity of the protozoan parasites was greater than that of helminths since seven species were identified in total but with lower prevalences (see Table 3). Species of at least three different genera of intestinal flagellates, i.e. *Hexamita* (0.007%), *Tritrichomonas* (0.004%) and facultative pathogenic flagellates such as *Hexamastrix* spp. and *Trimitus* spp. (0.001%), two different genera of amoeba [*Hartmanella* (0.001%) and *Entamoeba* (0.005%)], one ciliate genus *Balantidium* spp. (0.007%), and yeast-like *Blastocystis* spp. (0.002%) occurred in tortoise faecal samples. Microscopy did not reveal *Cryptosporidium* oocysts in any of the faecal samples. Although representatives of the ciliate genus *Nyctotherus* were commonly found in faecal samples, these were not quantified, since the species of this genus are considered non-pathogenic for tortoises and are found in high prevalences in herbivorous tortoises without clinical symptoms [12]. In a *T. hermanni* tortoise, we found high numbers of *Balantidium* trophozoites accompanied by facultative pathogenic bacteria (*Stenotrophomas maltophila* and *Morganella morganii*) and in a *Geochelone elegans* tortoise infected with *Balantidium* spp. clinical signs such as anorexia and severe debilitation were reported. Illustrations of selected parasite stages and histopathological findings are depicted in Figs. 2 and 3. Collected data (e.g. tortoise age, weight, gender and diagnosed parasites in the corresponding faecal sample) are provided in Additional file 1: Table S1.

### Necropsies

**Fig. 4:**
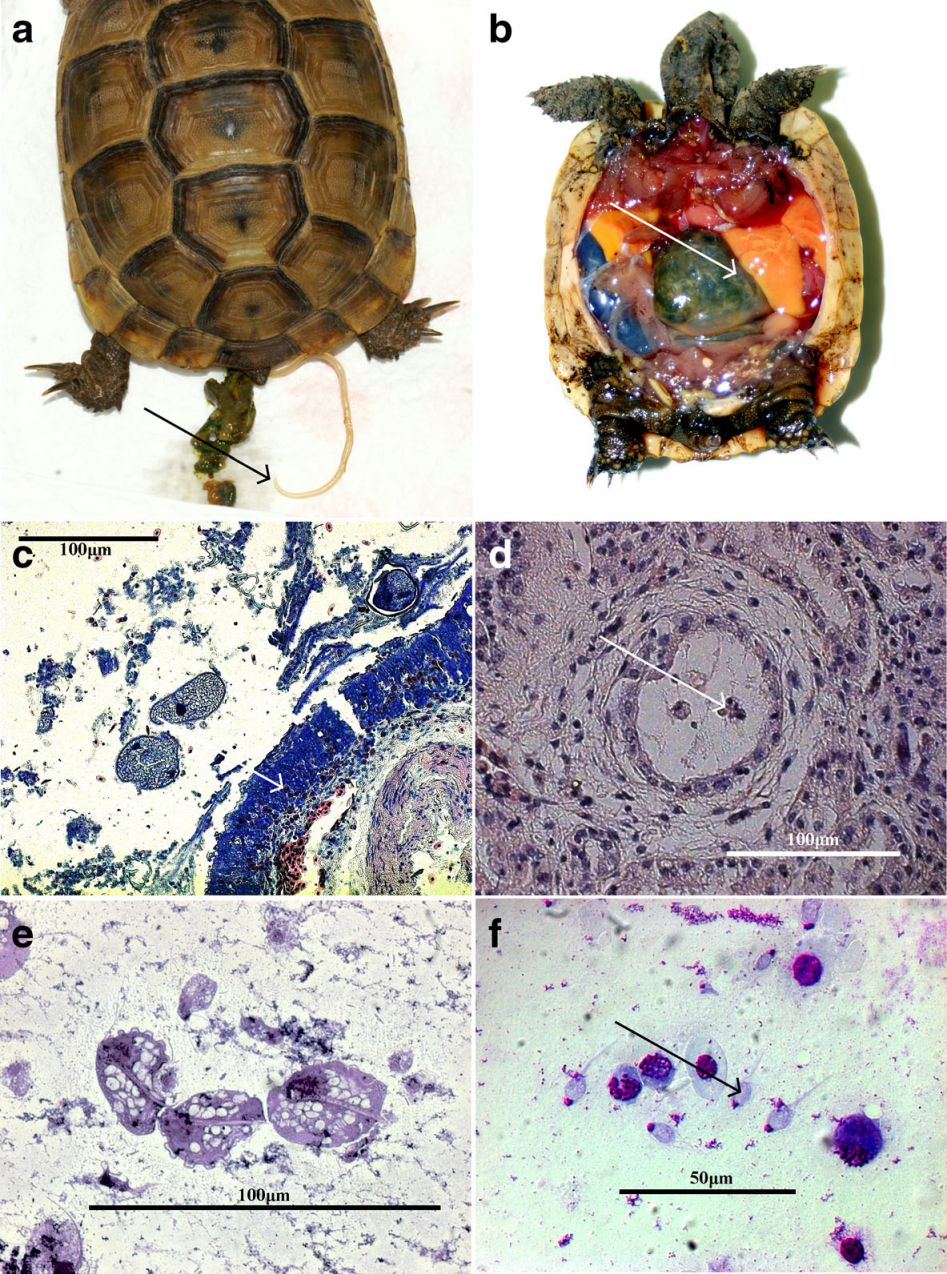
**a** Massive *Angusticaecum* sp. infection in a Hermann tortoise (*Testudo hermanni*) (leading to spontaneous expulsion of an adult nematode (arrow). **b** Lethal oxyurid infection in a necropsied juvenile tortoise (*T. hermanni*) with a massive colonic oxyurid infection (indicated by an arrow) associated with fatty liver degeneration (i.e. egg-yolk yellow liver). **c** *Balantidium*-associated enteritis (HE staining): free *Balatidium* sp. trophozites detected in the gastrointestinal lumen neighboring an eosinophilic-infiltrated (indicated by arrow) mucosa (*T. hermanni*, 400×). **d** Subchronic hexamitiosis (HE staining): interstitial nephritis with lymphocytic infiltration caused by *Hexamita* sp. Flagellated *Hexamita* trophozoites can be detected in the renal tubule lumen (indicated by arrow) of a Spider tortoise (*Pyxis arachnoides*,1000×). **e** Three trichomonad trophozoites with undulating membrane extending almost to their entire body length (Giemsa staining, 1000×). **f** Impression preparation of a kidney containing *Hexamita* sp. trophozoites with characteristic anterior protruded nuclei (arrow, Giemsa staining, 1000×)

In total, 49 individual tortoises from at least eight different species were dissected (for details see Table 2). Of these, 19 tortoises were adults, 25 animals were juveniles, and in 6 cases no age-related data were obtained. In total, 38 tortoises (77.6%) tested positive for endoparasite infections (Table 4), 12 tortoises (24.4%) died likely due to severe oxyuridosis, and two tortoises (4.08%) died of severe *Hexamita* spp. Infections. Other contributors to tortoise deaths were gout disease (*n* = 11; 22.45%), viral infections [Picornavirus (*n* = 6; 12.25%), Herpes virus (*n* = 3; 6.12%)], chronical nephrosis (*n* = 3; 6.12%), bacterial infections (*n* = 2; 4.08%), systemic mycosis (*n* = 1; 2.04%), cachexia and osteomalacia (*n* = 1; 2.04%), intoxication (*n* = 1; 2.04%) and sand obstipation (*n* = 1; 2.04%). In six tortoises (12.25%) the final death aetiology could not be identified (Fig. 4).

In total, 22 tortoises (44.9%) showed macroscopic and histological signs for metabolic bone disease (MBD), and 15 of these (68.18%) were infected with oxyurids (e.g. *Tachygonetria* spp.) and also showing signs for nephrosis/nephritis. Although the factors ‘oxyurid burden’ and factor ‘MBD’ correlated positive (Fisher’s exact test: *P* = 0.038), higher oxyurid burdens could not be identified as the only aetiological cause for MBD. An overview of isolated bacteria in combination with pathological diagnosis or reported clinical signs together with parasitic infection is given in the Additional file 2: Table S2.

## Discussion

Gastrointestinal endoparasites in pet tortoises have been investigated in several European countries [12–14, 16, 24, 38]. These studies report extremely high prevalence of oxyurid infections in tortoises kept in captivity. These findings were consistent with high oxyurid prevalence observed in our study (43.18%) and with our even higher prevalence recorded in dissected tortoises (57.14%). Oxyurids (pinworms) in tortoises are frequently reported [12–14, 16, 25, 38]. Studies at the generic level revealed the presence of *Tachygonetria*, *Thaparia*, *Mehdiella* and *Alaeuris* quite abundantly in tortoises of all ages [25, 27]. Oxyurid infections are proposedly tolerated and also to have rather low pathogenic effects on parasitized tortoises. The peculiar features of the large intestine of tortoises makes it plausible to assume that these nematodes have evolutionarily adapted to the tortoise gut microhabitat. Even an equilibrium of parasite-host-interactions has been postulated [28]. Moreover, oxyurids might be beneficial for certain tortoise species: some oxyurid-infected juvenile tortoises showed improved nutrient uptake and digestibility for most food components, since oxyurid nematodes might help to break up faecal masses, thereby preventing constipation [40]. Furthermore, oxyurids might contribute regulation of the bacterial flora in the caecum of herbivorous reptile hosts by feeding on bacteria [28]. However, massive oxyurid infections can lead to severe malabsorption including clinical symptoms such as anorexia and diarrhoea, impaction, chronic weight loss and even sudden death [12, 40]. Such oxyurid-derived pathogenicity is mainly linked to the monoxenous life-cycle and histiotrophic phase of oxyurid larvae, limited space for tortoises in captivity, low heterogeneity of oxyurids, and their ability to survive inside hibernating tortoises [12, 28, 41]. Juvenile tortoises in particular are affected by clinical oxyuridosis [28], since the life-cycle of oxyurids is completed within no more than 40 days. In our study, severe oxyuridosis was frequently (57.14%) diagnosed *post-mortem* in tortoises and was the predominant cause of death for many (24.49%) individuals included in necropsies. Moreover, oxyurid infections were often combined with other associated, factorial diseases such a liver degeneration, renal diseases (nephrosis/nephritis) or metabolic bone disease (MBD). Therefore, we advocate that oxyuridosis should be considered an important parasitosis under unfavourable husbandry conditions (i.e. inappropriate food supply, inadequate access to UV light, too dry conditions and insufficient maintenance hygiene) because of owners’ lack of knowledge, especially for juvenile tortoises (< 5 years) [12]. Since other endoparasitic infections, such as *Cryptosporidium* spp. and *Balantidium* spp. infections, can lead to enteral calcium malabsorption in tortoises, also infections with oxyurids in juvenile tortoises might result in severe calcium deficiency. However, although we found that MBD rate increased with oxyurid burden, we were unable to single out increased oxyurid burdens as the only aetiological cause for MBD. Seeing that most study animals were also diagnosed with nephritis or nephrosis in conjunction with MBD, a secondary renal hyperparathyroidism might be linked to MBD symptoms (Fig. 2c).

In this study, oxyurid infections were more common in young tortoises (< 5 years of age), than in adults (Table 5). Differences in oxyurid egg shedding in relation to tortoise age are poorly understood, and host immune responses, coprophagy or oxyurid species-specific effects might play a role in pathogenesis in juvenile tortoises [12, 28].

More samples were oxyurid-positive in summer (April-September) than in winter (October-March) (see Fig. 1b, c and Table 5). Reasonably, newly purchased tortoises with an unknown parasite status are frequently tested by the owners during spring/summer. Also, after diagnosis of oxyuridosis, an inefficient deworming, inadequate quarantine, false negative faecal samples or even no confirmation of efficient deworming before entering hibernation period could explain our findings.

In contrast to oxyuridosis, prevalences of ascarid infections, most probably *Angusticaecum holopterum* [16, 24]*,* in sampled tortoises were extremely low (*n* = 12; 0.01%,). Half of these ascarid-positive animals were co-infected with oxyurid species (*Proatracis* sp. Was found in one necropsied tortoise). Ascarid prevalences of 0.01% in our study were very low compared with those reported in other Europe-based tortoise studies which reported prevalences between 8.5–28.0% [12, 16] and even up to 56.9% [13] for tortoises maintained in captivity. For Germany, an ascarid prevalence of 1.5–2.7% in captive tortoises was previously reported [12] which is still higher than reported in the current study. Very low prevalences of ascarid infections observed in our study might be linked to diagnostic technique used here. Methods used in other studies combining flotation, the McMaster technique, and/or SAF (sodium acetate acetic acid formalin) methods might thus be more appropriate diagnostic tools to detect ascarid infections in reptiles [3, 13]. In addition, irregular and self-consistent, veterinary-independent anthelmintic treatments and/or inadequate hygiene conditions of owners might have influenced ascarid prevalences. In this study, the youngest ascarid-infected tortoise was two years of age and the oldest ten years, indicating that ascariosis likely affects all age groups of tortoises. Also, we found one ascarid-positive animal, a juvenile leopard tortoise (*Stigmochelys pardalis*), which showed an unusual gastrointestinal flora while the presence of potentially pathogenic microorganisms such as *Acintobacter* sp. and *Aspergillus* sp*.* [42–44] were diagnosed. Currently, species of the ascarid genus *Angusticaecum* in tortoises seems more pathogenic than oxyurids since the life-cycle of *Angusticaecum* spp. includes larval migration and further development in various organs, and is associated with anorexia, exsiccosis and weight loss [14, 16]. Eggs of *Angusticaecum* spp. have a three-layered shell and a sticky external mucopolysaccharide chain layer, which makes these eggs adhesive to the surface of environmental structures and resistant to commonly used disinfectants. Due to the high tenacity of *Angusticaecum* spp. eggs, the management or even eradication of *Angusticaecum* infections in tortoises can thus become a major exhausting challenge.

The low prevalence of *Strongyloides* spp. eggs in our study is in contrast with Ratai et al. [13], who reported a *Strongyloides* prevalence of 3.7% in necropsied tortoises. Only one animal was infected with heterakids (0.001%) which was much lower than prevalences published elsewhere [12]. Heterakid nematodes of the genera *Meterakis* and *Africana* are known to parasitise in Testudinae [45]. Heterakids do not commonly parasite in herbivorous tortoises, but in more-insectivorous hosts such as chameleons and geckos, as well as insectivorous tortoises of the genus *Kinixys* [45, 46]. Since numerous heterakid species do not show strict host specificity [46], transmission to herbivorous tortoises might be possible when keeping them together with insectivorous reptile species, for instance in zoos.

In contrast to helminths, much less is known about occurrence and pathogenicity of intestinal protozoans in captive reptiles [7, 10]. Species of most intestinal flagellate genera such as *Trepomonas*, *Trimitus*, *Chilomastix* and *Retortamonas* are not considered pathogenic, or their pathogenicity is still unknown [7, 12]. Conversely, the enteroflagellate *Hexamita parva* has been described as a pathogenic species due to its tendency to ascend towards organs connected to the gut, such as the kidneys, urinary bladder and liver [6]. Consistently, *Hexamita-*infection was the second-most frequent parasitosis found in this study. More importantly, hexamitosis occasionally resulted in fatal outcomes, thus supporting previous reports of its high pathogenicity [6, 12]. In our epidemiological study, mortal hexamitosis was only surpassed by oxyuridosis, indicating the significance of hexamitosis in pet tortoises in Germany. Chronic hexamitosis in chelonian tortoises can result in severe tubular-interstitial nephritis, granulome formation, tubular necrosis, mineralization, gout, hyaline casts and even fibrosis [6, 12]. The high pathogenicity of *H. parva* presented in this study calls for more investigations on this neglected intestinal parasite.

We also identified potentially pathogenic protozoans (i.e. *Tritrichomonas*), and facultative parasites (i.e. *Blastocystis*) in pet tortoises [10, 37]. *Blastocystis* infections are often found in clinically healthy reptiles [37]. Moreover, due to its rather high prevalence in herbivorous tortoises without clinical symptoms [12], the ciliate genus *Nyctotherus*, with its species all are regarded to be apathogenic, was also identified in our study but not quantified. In contrast to *Nyctotherus* spp., *Balantidium* spp. can induce enteritis in heavily infected animals or by acting synergistically in association with other parasites [12, 14]. Confirmatively, one necropsied tortoise showed a severe *Balantidium*-derived colitis with a concomitant oxyurid infection. Balantidiasis in tortoises has generally been reported to be mostly non-pathogenic [14, 16]. Nevertheless, we note that one *Geochelone elegans* tortoise that displayed anorexia and severe debilitation, was also infected with high numbers of *Balantidium* trophozoites. In the colon of one necropsied *T. hermanni* tortoise, we found high numbers of *Balantidium* trophozoites accompanied by facultative pathogenic bacteria (*Stenotrophomas maltophila* and *Morganella morganii*) [42–44] evidencing the pathogenic potential of *Balantidium*.

The amoebae of the genus *Hartmanella* might infect tortoises kept in captivity but until now are still considered non-pathogenic parasites [7]. In contrast, other amoeba genera, such as *Entamoeba*, with its pathogenic species *E. invadens*, should be considered pathogenic since they can cause anorexia, watery diarrhoea and epizootic amebiasis in herbivorous tortoises [7]. As a general recommendation, infections with these parasites should always be considered in routine reptile diagnosis by veterinarians, particularly when corresponding clinical symptoms are present.

Overall, we found neither eggs of cestodes, trematodes or pentastomids nor oocysts of *Cryptosporidium* spp. Since reptiles act as definitive hosts for pentastomids, special attention for ‘One Health’ reasons should be paid to these parasitic crustaceans. Some pentastomid species have anthropozoonotic potential and are largely unknown to the scientific community [8]. According to the indirect the life-cycle of pentastomids, only carnivorous or piscivorous chelonians should be affected and act as definitive hosts [8]. Our large-scale study (*n =* 1005) suggests that herbivorous tortoises held in German private homes or zoos are generally not infected with pentastomids. Herbivorous tortoises thus likely play a neglectable role in human pentastomid-derived infections.

While we did not find any oocysts of *Cryptosporidium* in this study, cryptosporidiosis occurs in tortoises, manifesting in classical enteritis symptoms such as chronic diarrhoea, weight loss and/or unusually soft faeces due to maldigestion [47–49]. A recent study from the Czech Republic showed a *Cryptosporidium* prevalence of 11% in pet tortoises using microscopy- and PCR-analyses (*n =* 387) [50]. Saline faecal smears performed in the present study might have missed detection of minute *Cryptosporidium* oocysts (*c.*5 μm in size), thus the actual cryptosporidiosis prevalence might have been higher. Additionally, galled tree pollen in the faeces of free-ranging reptiles can cause false-positive results when saline smears are used for *Cryptosporidium* oocyst detection. Further epidemiological studies on cryptosporidiosis in pet tortoises are required using more sensitive methods such as carbol-fuchsin faecal smears, the usage of commercially available copro-ELISAs (e. g. ProSpecT® *Cryptosporidium* Microplate Assay), direct immunofluorescence (Meriflour®), or even *Cryptosporidium*-specific copro-PCR as previously proposed [50]. The zoonotic potential of reptile-derived *Cryptosporidium* species has recently been discussed [51, 52]. Until now, neither anthropozoonotic *C. parvum*/*C. muris* infections have been identified as transmissible to reptiles, nor have *Cryptosporidium* infections from reptiles to endotherms been demonstrated under experimental conditions to date [53, 54].

## Conclusions

The infection of several pathogenic parasite species found in our epidemiological study calls not only for a faecal examination of tortoises prior to being introduced to a new owner, animal group, or enclosure, but also shows that endoparasite health screenings should be performed and, if diagnosed, appropriately medicated exclusively by veterinarians. Animal welfare and hygiene issues in particular, as well as illegal pet trade issues, demand that routine coprological diagnosis of parasitoses are recognised as a paramount aspect of reptile medicine. Based on this parasitological study, we advocate for further and more detailed research on this still neglected herpetological issue.

## Additional files

Additional file 1: **Table S1.** Data obtained from necropsies and faecal samples such as date of receive, post code and information collected from pet owners such as age, husbandry conditions, gender, length, weight, species and diagnosed parasites. (XLSX 394 kb)

Additional file 2: **Table S2.** Isolated bacteria in pet tortoises from Germany; origin and species of tortoises regarding to the infestation with potentially health-critical endoparasites, performed microbiology and aetilogical death reason/ reported clinical signs. (DOCX 18 kb)

## Abbreviations

ELISA: Enzyme-linked immunosorbent assay
MBD: Metabolic bone disease
