# Supplementary material for: Occurrence of health-compromising protozoan and helminth infections in tortoises kept as pet animals in Germany
Source: Parasit Vectors. 2018 Jun 18;11:352. doi: 10.1186/s13071-018-2936-z (PMC6006665; doi:10.1186/s13071-018-2936-z)
Supplement: Supplementary file 2 — Table S2. Isolated bacteria in pet tortoises from Germany; origin and species of tortoises regarding to the infestation with potentially health-critical endoparasites, performed microbiology and aetilogical death reason/ reported clinical signs. (DOCX 18 kb) [file 13071_2018_2936_MOESM2_ESM.docx]

**Additional file 2: Table S2** Isolated bacteria in pet tortoise from Germany

**_____________________________________________________________**

**Case No / tortoise species origin sample parasitic infection isolated bacteria / location clinical signs / aethiological death reason_______**

1. *Testudo hermanni* private feacal sample - *Pseudomonas orzyihabitans* (+++), *Ps. putida* (++),

*Candida* sp. (++) -

1. *Centrochelys sulcata*  private fecal sample - *Stenotrophomonas maltophila* (+++), *Acinetobacter* sp. (++), -
2. Unkown private feacal sample - *Enterobacter faecalis* (++), *Bacillus* sp. (++) -
3. *T. hermanni*  private feacal sample Oxyurids (++) *Citrobacter* sp. ++, *Acinetobacter* (+) *Proteus vulgaris* (+++) -
4. Unknown private feacal sample - *C. braakii* (++), *S. maltophila* (++), *Mucor* sp. (++), -

*Acinetobacter* sp. (+)

1. *Stigmochelys pardalis* private feacal sample Ascarids (+) *Acinteobacter junii* (++), *Aspergillus* sp. (++) -
2. *T. hermanni* private section Oxyurids (+++) no bacteria / coeloma ascites, degeneration of liver/kidney, wormileus
3. *T. hermanni* private fecaal sample - *Citrobacter* sp. (++), *Acinetobacter* sp. (++) -
4. *T. horsfieldii* private section - *Klebsiella pneumoniae* (++), *Pseudomonas flourescens* (++) / coelom bacterial infection, lung edema, hepatitis, nephritis, MBD
5. *T. hermanni* private section Oxyurids (+++) none / coelom heavy Oxyurid infection, epithelium degeneration, enteritis, nephritis, generalized edema, MBD
6. *T. hermanni* private section Oxyurids (+++) none / coelom heavy Oxyurid infection, epithelium degeneration, enteritis, nephritis, generalized edema, MBD
7. *T. hermanni* private section Oxyurids (+++) none / coelom heavy Oxyurid infection, enteritis, nephritis, MBD
8. *T. hermanni* private section Oxyurids (+++) none / coelom heavy Oxyurid infection, epithelium degeneration, enteritis, nephritis, gout
9. *T. hermanni* private feacal sample Oxyurids (++) *Citrobacter* sp. (+++), *S. maltophila* (++), *Mucor* sp. (++)
10. *T. hermanni* private feacal sample Oxyurids (+) *Mucor* sp. (++), *Enterobacter* sp. (+++), *Ochrobactrum antropi* (++) -
11. *T. hermanni* private section Balantidium sp. (+++) *S. maltophila* (+++), *Morganella morganii* (++) / coeloma bacterial infection of live, hearth, kidney and intestines, generlized edema, MBD
12. *T. graeca* private feacal sample - *Citrobacter* sp. (++), *E. faecalis* (+++) -
13. *T. horsfieldii* vet feacal sample - *Acinetobacter* sp. (+) -
14. *T. horsfieldii* vet feacal sample - *Citrobacter* sp. (++) *Acinteobacter* sp. (+++) -
15. *T. graeca* vet section - *Mucor* sp. (+++) / coelom systemical mykosis, glossitis, enteritis, hepatitis/nephrosis
16. Unknown vet section Oxyurids (+),

*Blastocystis* sp. (+++) none / coelom gout, urate in pericardial sack, hepatosis

1. *T. hermanni* vet feacal sample Oxyurids (++),

*Hexamita* sp. (++) *Mucor* sp. (++), *Acinetobacter* sp. (+++), *Ps. orzyhabitans* (++), -

*Ps. stuzeri* (++) / intestinal content

1. *C. sulcata* vet feacal sample - *Ps. orzyhabitans* (++), *Ps. stutzeri* (++) -
2. *Geochelone elegans* zoo feacal sample *Blandtidium* sp. (+++) *E. feacalis* (+++), *Proteus vulgaris* (+++) apathy, anorexia
3. *Stigmochelys pardalis* private section Oxyurids (++), Heterakids (+++) *Stenotrophomonas maltophila* (+++) / coelom Enteritis, nephrosis, Hepatosis
4. *G. chilensis* private section - *Proteus vulgaris* (+++), *Bacillus* sp. (++) / coelom Pneumonia, Enteritis, hepatitis, virus x suspition
5. *T. hermanni* private section Oxyurids (+) *S. maltophila* (+++), *Mucor* sp. (+++) / skin fungal necrotical dermatitis, myocarditis, kidney gout
6. *T. horsfieldii* private section Oxyurids (+++) none / coelom generalized edema, wormileus, nephrosis, hepatosis
